# The negative effects of social media on the social identity of adolescents from the perspective of social work

**DOI:** 10.1016/j.heliyon.2021.e06327

**Published:** 2021-02-21

**Authors:** Walaa Elsayed

**Affiliations:** College of Humanities and Science, Ajman University, Ajman, United Arab Emirates

**Keywords:** Negative effects, Social media, Social identity, Adolescents, Social work

## Abstract

The aim of this study was to understand the social identity levels of adolescents and to analyze the negative effects of social media on their social identity from the perspective of social work. The researcher used a descriptive-analytical technique in this study. The study's sample consisted of 200 adolescents (male and female) in the secondary stage at age group (15–18 years). The researcher designed a questionnaire based on the four main levels of James Marcia's theory of social identity. The results showed a variety of negative effects of social media on the social identity of adolescents in terms of "achievement - postponement - closure - dispersion", this requires taking serious measures from the family, the school, and other institutions to care for the family and the child to strengthen how to face these risks to protect the identity of adolescents from violating their privacy and negatively affecting their intellectual principles.

## 1 Introduction

Adolescence is the stage of cultural and social formation, it is the most critical juncture for children and youth. If there is no guidance, care, and follow-up from the adolescent's family and his school, the adolescent, in his quest to develop a sense of social identity, spends most of his time thinking, reviewing, and reflecting on the general values and behaviors he observes (Bakkar, 2010, p55). He must decide how to succeed in friendships with his peers, exercise his social roles as appropriate, and choose between multiple beliefs, ideas, and options that will give him a sense of distinct and independent existence working towards building his own future. In this light, adolescents are exposed to what is known as an identity crisis (Levesque, 2011, p.109, p.109).

The crisis of social identity is the main problem people must tackle during adolescence. The crisis starts with the beginning of the formation of a personality where the adolescent asks a number of questions to himself such as: Who am I? What is my role in society? How do I prove my existence? How do I succeed? Here, the adolescent finds himself faced with multiple questions, contradictory demands, and ideas, which force him to deal with multiple conflicts, especially in light of physical, mental, social, psychological, emotional, and family changes. If these changes are negative, it will result in the failure of the adolescent to successfully form his identity, in addition to facing many problems such as social role disorder, identity confusion, or the adoption of negative identity, harming the adolescent's life and future (Salima and Fayza, 2014. p.384).

### 1.1 Definition of social identity from the perspective of social work

The thinker Alex Mitchell considered that identity is an integrated system of physical, psychological, moral and social data involving a pattern of cognitive integration processes (Mitchell et al., 2016, pp12-16). It is characterized by its unity, which is embodied in the inner spirit, and has the characteristic of the sense of identity and intelligence (Mitchell et al., 2016, pp127-138). Identity is the unity of internal feelings (Asiri, 2004, p.122), which is the unity of physical elements, differentiation, permanence, and central effort. This means that identity is a unit of integrated physical, social and psychological elements, which makes a person distinct from others, and enables him to feel his own unity (Dawaq, 2016, p.26).

Erik Erikson believes that the identity of an individual is formed during a long struggle, which begins in adolescence, and focuses on the composition of two element (Erikson, 1994, p15). The first is the acquisition of the ability to create a relationship with the surroundings, and the second a sense of integration into a suitable moral world. We believe that both elements are necessary and complementary to each other because the individual needs to identify himself within his society. When people ask us who we are, they do not usually mean the name we carry, but our position in the social network, that is, the small circle that we belong to within the great social circle, and the job that we do within this circle (Salima and Fayza, 2014, p.390). Therefore, the individual does not just mention his first name, but adds to him the last name, and then the functional definition, which refers to occupation, hobby or status. This leads us to the second element, namely, the need for an individual to have a meaningful world in which to enjoy his or her abilities and receive the appropriate reward for what he does. This is why all people seek to build relationships with a group, because much of the pleasure of life, or happiness, is achieved through interaction between individuals. Hence, it is said that those who dispense with people lose the sense of beauty of life (Al-Shammasi, 2006, p.23) because in fact they lose the need for the daily challenge posed by the physical and moral interaction between the individual and his environment (Cillessen and Rose, 2008, p.143). It is the need for integration that imposes on the individual a pattern of personal choices and descriptions that may not necessarily be the best for them but are necessary to make their way into the community. On the other hand. groups may vary in their susceptibility to the integration of new individuals, in the sense that they may set difficult conditions, require the individual to give up his own choices in return for enjoying the virtues of social living, or simply refuse to integrate any new individual unless they are fully identical with them in psychological and social growth (Al-Hafian, 2004, p.30).

### 1.2 James Marcia's theory and levels of social identity

The theory of Marcia is based on a significant assumption that a well-defined and independently determined identity exists for the mature and well-adjusted person. This presumption expresses an implicit collection of shared principles, with a putting great emphasis on human interests, rights, and freedoms. Therefore, maturity in terms of a highly developed sense of an individual self is only natural, and maturity is characterized by the willingness to subjugate individual pursuits and desires in the service of the greater good of the group (Morelli, 2020, pp.12–24).

There are four key points or milestones that James Marcia's theory has descriptively defined along the continuum of identity growth. Such stations or points describe very different states of identity, ranging from a diffuse and indeterminate individual identity to a precise and extremely specific individual identity. Marcia assumed that such conditions and events (called 'crises') act as catalysts for movement along this continuum and through the different status of identity. These crises cause internal tension and emotional upheaval, forcing teenagers to analyze their values, beliefs, and aspirations and doubt them. They can develop new beliefs, accept different values, and make different choices as they explore new possibilities. Every identity status is a basic configuration of the progress of an adolescent with regard to identity exploration and dedication to the values, beliefs, and goals that contribute to identity (Marcia, 1966, p 551), Marcia used the concept of identity status to identify four stations or points of unique developmental identity as follows:

Social identity achievement: This status of identity reflects both a high degree of experimentation and a high level of dedication. It is said that teenagers have achieved their identity through an active discovery phase and a deep commitment to a clear set of values, beliefs, and life goals that have resulted from this active exploration and analysis. Adolescents will have determined what ideals and priorities are most important to them at this identity status, and what purpose or task will drive their life. individuals at the status of identity achievement may prioritize what is relevant to them and have sorted who they want to be by the many possibilities. They would have experimented and examined their journey in life with several different convictions and values. Young people need to feel optimistic and secure in their choices and beliefs to truly achieve this form of identity (Marcia James, 2011, p101). In addition to achieving a goal as a result of the individual's experience after a temporary period of exploration, including testing values, beliefs, goals, and roles, selecting what was meaningful or personal and of social value, and then demonstrating a true commitment to what was chosen to implement it (Al-Ghamdi, 2001, p.86).

Social identity postponement: This identity status reflects a high level of experimentation but a low degree of dedication. At this point, teenagers are in the midst of a crisis of identity that has prompted them to explore and experiment with various values, beliefs, and goals. They have not, however, made any definitive decisions as to which principles and beliefs are most important to them, and which values should guide their lives. Therefore, they are not committed to a specific identity yet. They keep their choices and alternatives open (Marcia, 1966, p 550). In addition to continuing to try and test the available options without reaching a final decision and without making a real commitment to specific options, which causes the individual to change his choices from time to time in an attempt to reach what is appropriate (Abu Arad, 2008, p.18), including but not limited to changing the field of study, profession, identities or friends (Steinberg, 2002, p.33).

Social identity closure: This status of identification indicates a low level of discovery but a high degree of dedication. Adolescents do not consciously seek to decide what is important to them in this identity status. The principles and beliefs they have been taught are not questioned. Instead, by clearly embracing the ideals and values of their families and community culture, these teenagers obtain their identity. In a way, the personality given to them is passively embraced by them. Although these young people are committed to their assigned ideals and life goals, they do not ask why they should be, nor do they suggest any alternatives (Marcia, 1980, pp.159–187), in addition to their avoidance of any subjective attempt to reveal beliefs, goals and social roles of meaning or value in life, but they are contented with satisfaction of the roles as determined by external forces such as family and society (Al-Zu'bi, 2001, p.477).

Social identity dispersion: This identity status describes adolescents who have neither explored any real identity nor committed to it. This status of identity thus reflects a low level of experimentation and a low level of dedication. These teenagers have not at all considered their identity, and have not set any goals for life. They are reactive, floating through life passively, and dealing with every situation as it arises. Their main motivation is hedonism, avoiding discomfort, and gaining pleasure (Marcia James, 2011, p101), in addition to the lack of individual sense of the need to form a philosophy, goals, or specific roles in life, on the one hand, with the absence of commitment to the roles which led by chance on the other. This happens with the aim of avoiding the individual researching and testing to preferring compatibility with problems or solving them by postponing and disrupting (Khader, 2018, p.89).

In light of the above, the individual's identity is formed solely by the interaction of the individual with others, and the individual's view of others is partly shaped by the way others view that individual. According to the theory of symbolic reactivity (role theory) (Al-Murshidi, 2007, p.27), people continue to possess their individuality but are not entirely distinct from society (Ali, 2007, p.83), and identity acts as a bridge between the individual and those around him (Mohsen, 2018), for this reason, we must work hard to monitor and follow up our children in their way of life especially after the recent boom in electronic means of communication and the spread of social media which has become a remarkable presence all over the world, especially among children and young people and despite the positive effect of some social media, but the social media can also have a destructive influence on social relations between adolescents and their families, in addition to the negative effect on the academic achievement of adolescents.

### 1.3 Definition of social media

The phenomenon of social media began in 1997, and the site "Six Degrees.com" was the first of these sites providing the opportunity for users to create profiles, comment on news, and exchange messages with other participants (Mohamed, 2019, p.6). Although the site "Six Degrees.com" is the pioneer of social networking, "My Space.com" has opened wide horizons and achieved tremendous success since its inception in 2003 (Hayaty, 2018, p.3). Then successively began the emergence of social media, but the milestone is the emergence of ‘Facebook.com’ which enables users to share information among themselves and allow friends to access their profiles (Al-Shareef, 2014, p73), for this reason, the use of various social media has become a daily occurrence in modern times (Mashaal, 2018, p.56).

Some scholars define social media as virtual places where communication through the means of dialogue, chat, comment, photography, and interaction between users can take place without borders or breaks (Al-Jazi, 2018, p.14). So, the internet is described as a virtual space because it is considered a liberating place where no one party owns it (Asur and Huberman, 2010, p.40), and defines social media as services that are created and programmed by major companies to gather the largest number of users and friends who share activities and interests, searching for more friendships and the interests and activities of other people with whom they share one of the intellectual contributions (Bailey et al., 2009, p. 10 & Salim, 2008, p.56). These social media provide features such as instant messaging, public and private messaging, and multimedia sharing of voice, video, image, and files (Rajah, 2019, p.86), which has attracted millions of users from around the world. (Mansour, 2014, pp.287–288), and also social media are an electronic social structure made from individuals, groups, or institutions, the basic composition (such as an individual) of which is called a term (node) where these nodes are connected to different types of relationships (Al-Mu'ti, 2016, p.93). Such as supporting a specific sports team, belonging to a company or nationality of a country in the world and these relationships may reach deeper degrees (Ali, 2019, p.102), such as social status, beliefs, or class to which the person belongs (Salima and Fayza, 2014, p.391), and there are many types of social media used by children and adolescents such as Facebook, Instagram, Twitter, Snapchat, WhatsApp, etc (Hamed, 2018, p.7).

### 1.4 Advantages for children and adolescents of using social media from the perspective of social work

Provide the opportunity to connect with friends, family, and colleagues who share the same interests, and share pictures, ideas, and fun moments with each other (O'Keeffe and Clarke-Pearson, 2011, p127).

Provide an opportunity to join in community service projects through what is known as “e-volunteering”.

Developing individual and collective creativity through exchanging technical projects and benefitting from innovative experiences (Muzayd, 2012, p.42).

Promoting educational opportunities developing ideas and raising intelligence through the creation of blogs, videos, and game sites (Ito et al., 2008, p.15).

There are ways to regulate and control privacy and confidentiality rules not based on reparation or compulsion but rather on choice, and users can block or report inappropriate or unacceptable interventions and materials.

Provide an opportunity to learn, to exchange respect, tolerance, and constructive dialogue on global humanitarian issues to promote human identity and social skills (Abdul Jalil, 2011, p.247).

### 1.5 Risks for children and adolescents of using social media from the perspective of social work

Threat and harassment through the Internet: through the dissemination of false information, embarrassing or hostile interaction from others. This is one of the biggest risks of using the internet for adolescents, it is a risk from peer to peer and can cause profound social and psychological consequences such as depression, anxiety, isolation, and tragic suicide (Nomar, 2012, p.193).

Send sexual messages (sexting): through the sending and receiving of sexual messages and images through mobile phones, computers, and other digital receivers where images become rapidly spread via mobile phone and the internet. This phenomenon can be seen in recent research which has shown that 20% of adolescents published pictures of their own showing themselves naked or semi-naked, with some of them having been accused and convicted on charges of felony publishing porn (AL-Oubli, 2011, p.826).

Facebook depression: which occurs in adolescents as a result of spending a lot of time on social media sites such as Facebook and then beginning to show symptoms of depression through social isolation from their environment and their families, with some resorting to using dangerous sites and blogs, which may promote addiction or sexual relations and/or destructive, self-aggressive behaviors. Social media sites lead to the isolation and destruction of family relations (Hosni, 2011, p. 101).

The collapse of the idea of the reference group in its traditional sense. The virtual society is not determined by the place, but by the common interests that bring people together, who did not necessarily know each other before meeting electronically. They are sleepless societies; one can find a contact with another around the clock. Virtual societies are highly decentralized and gradually result in the dismantling of the concept of traditional identity. The disintegration of identity is not confined to national or resident identity, but also to personal identity, because those who use social media often use pseudonyms and avatars, and some have more than one account (Al-Obaidi, 2019, p.18).

Digital Footprint and Privacy concerns: This is related to the lack of privacy for adolescents, due to a lack of experience in the safe use of social media sites, who exchange a lot of private information or disseminate false information related to them or others putting their privacy at risk. In addition, the presence of the property is collected and user information recorded on the internet resulting in something called a "digital fingerprint." (Zain Al Abdeen, 2013, p.2).

### 1.6 Previous studies

Several studies have indicated that one of the most significant difficulties experienced by adolescents is a conflict of values linked to their continuous search for identity and belonging (Bouchey and Furman, 2013, p.319). This is compounded with a desire to achieve self-direction by going into the unknown; interacting with strangers on social media sites and entering into a network of virtual relationships via the internet. This corresponds with the study (Laith, 2011) on “The Impact of Using Social Media site "Facebook" on Youth Self-Esteem” which demonstrated the role Facebook plays in modern upbringing through providing a platform for children and young people to discover ideas and convictions that greatly shape their future character values and determine their life trends. Traditional upbringing institutions lack the ability to monitor new behavioral patterns resulting from friction with the outside world caused by social media. The study also noted that a large number of young people have become isolated from their communities, hiding behind computer screens to connect with the virtual community instead. The study recommended the need to regulate the method and hours of social media use, while determining the quality of permitted sites and programs, and considering the increasing need for periodic supervision on children by their families. In this context, according to recent statistics, 22% of adolescents access their favorite social sites more than ten times a day and more than half of adolescents enter these sites more than once a day 75% of adolescents have a mobile phone, 25% of them use their phones to access these sites. and 54% use it to send SMS, whilst 24% use it for instant messaging. Thus, much of the social and emotional development of this generation takes place online via mobile phone (Zain Al Abdeen, 2013, p.2). And study (Safar, 2017), entitled “The role of social networks in the consolidation of the values of citizenship from the perspective of the Omani youth,” This study aimed to identify the role of social networks in establishing the values of citizenship from the viewpoint of the university youth in Oman, The study used the descriptive-analytical approach and relied on the questionnaire tool, It was applied to a random sample of 477 students from Sultan Qaboos University, The study concluded several results, including The social networks, reinforced the value of brotherhood among citizens and emphasized the cohesion Patriotism among community members, Social networks were used to promote solidarity, cooperation and assistance to the needy, The results showed that many social media sites were the most used among the sample members They are in order Facebook, WhatsApp, Twitter. And study (Hamdi, 2018), University Youth Dependence on Social Media for Access to Information, “A Survey Study at the University of Tabuk, Saudi Arabia”, The main objective of the study is to know the degree of dependence of Saudi youth on the communication sites Social In knowing the information and news they are looking for, and the study relied on the descriptive method and the questionnaire tool, It was applied to a sample of 401 students from the University of Tabuk in Saudi Arabia. The most important results of the study were the most important motives for the use of social networking sites by Saudi youth is entertainment and leisure time, then get news and information, then for social relations with friends and relatives.

Several studies have indicated there are the effects of social media on an individual's life, his academic achievement, and his progress in life such as study (Awad, 2013) entitled: “The effects of the use of social media sites on the educational attainment of children in Tulkarm governorate from the point of view of housewives”, which stressed the importance of the role played by mothers of teenage children who use social media sites. The study reached the important conclusion that social media sites have a negative impact on the educational attainment of children, especially in cases where mothers worked more hours. Therefore, it is necessary to target mothers with awareness campaigns and workshops, to raise the level of awareness of how they can monitor their children's use of these sites, and advise them of the need to establish rules and controls to monitor banned and destructive sites, so users cannot access pornographic sites. And study (AL-Aag, 2013) entitled: The use of the Internet in the study and its relationship to motivation for learning in adolescents (12–14 years). The purpose of this study is to explore the relationship between the use of the Internet and motivation for learning among teenage pupils, the researcher used the descriptive method, and relied on a simple random sample, the sample size represents 110 pupils. One of the most important results of the study, the proportion of males who use the Internet is estimated at 50% of the total sample, while the percentage of students who are highly motivated to learning 91.81%, and students with low motivation to learning 9.19%. And study (Hattat, 2014), entitled (psychosocial problems of school-age adolescents internet users), This study aimed at detecting the prevalence of psychosocial problems in terms of internet addiction, social isolation, lack of concentration, and depression in a sample of adolescents studying “internet users in Ouargla”, the sample of the basic study consisted of 406 students using the internet who were chosen intentionally among the students studying during the 2013–2014 school year, one of the most important results of the study was that the prevalence of psychological and social problems was low, where the percentage of Internet addiction was 2.95 %, 0.73% for social isolation and 2.70% for the problem of alienation. And study (Zawana, 2015), entitled ‘The degree of using social networks as a tool for learning among Jordanian university students and the achieved satisfaction’, the aim of this study was to investigate the degree of use of social media by Jordanian university students as a learning tool, the study used the descriptive approach in the field survey of the study community consisting of the University of Jordan as a public university and the Universities of the Middle East and Petra as private universities, the questionnaire was applied to a total sample of 400 students, they were asked closed questions on the five axes of the tool on the degree of use and the satisfaction of saturation, the results include: YouTube ranked first, followed by Facebook and Twitter respectively, students resort to the university's website in the first degree to learn the dates of the quarterly and monthly tests. In addition to studying (Bu-Abdullah, 2016), entitled " Internet uses and their impact on students Adolescents "Secondary Field Study of Khadr Ramadan Omash-Biskra", this study aimed to identify the uses of the Internet and its impact on teenage pupils and this study is descriptive, and selected the sample was random, Where included 26 students in high school, the questionnaire was used as a study tool, the study reached the following results: It was emphasized that the use of the internet leads to delayed level of academic achievement in adolescent pupils, the availability of the Internet inside the home increases the duration of use of the teenager, it was also emphasized that parents should know the programs which watching their teenage child is on the Internet to guide to the useful things on this network. And also study (Hinnawi, 2016), entitled " Uses of Middle Teen Students for Networks Social networking in Nablus city schools in Palestine, this study aimed to investigate the reality of the use of students adolescence to social networking, the study used the descriptive method and the sample of the study was 217 singles, one of the most important findings of the study is that the majority of students have at least one subscription on social networking sites by 97%, And 63% of them use smartphones as the main device in the use of social networks. And study (Kehinde and Adegbilero. 2016), entitled "Use of social media by science students in public universities in Southwest Nigeria", this study aimed to identify the extent of the use of social media in academic activities by students of the State University in southwestern Nigeria, The study was based on a descriptive curriculum and a purposive sample of 140 students from three educational institutions in southwestern Nigeria, the results of the study indicated that the students are a user of social networking sites in high rates, with 93.48% use Facebook, then Google by 63.77%, In addition, two-thirds of users use it for staying informed about events/news, then for leisure and entertainment, the most important obstacles facing them in the use of social networks are receiving unsolicited messages and power outages.

### 1.7 The present study and it questions

The present study is concerned with studying the negative effects of social media on the social identity of adolescents in terms of "achievement - postponement - closure - dispersion" from the perspective of social work, and this is a new aspect not addressed before. In order to do so, this research asks the following research questions:

Q1: What are the negative effects of social media on the level of "achievement" in the social identity of adolescents from the perspective of social work?

Q2: What are the negative effects of social media on the level of "postponement" in the social identity of adolescents from the perspective of social work?

Q3: What are the negative effects of social media on the level of "closure" in the social identity of adolescents from the perspective of social work?

Q4: What are the negative effects of social media on the level of "dispersion" in the social identity of adolescents from the perspective of social work?

Q5: Does the degree of awareness adolescents of the negative effects of social media on levels of their social identity vary according to gender, adjective, age, number years of using, and favorite app?

## 2 Methodological procedures

### 2.1 Sample

The study community is represented by all adolescent students in the secondary stage, an estimated (3836) students "males - females". As for the research sample, the sample was chosen randomly. The researcher used a simple random survey (a representative sample of the study population of adolescent students in secondary school), and the sample size was estimated (200) "males - females" adolescent student. The researcher used Cochran's Equation to calculate a sample size as shown in Eq. (1) (Cochran, 1963, p.75, p.75):

(1) $$n=\frac{n_{0}}{1+\frac{\left(n_{0}-1\right)}{N}}$$

Where **n** is denotes the sample size in limited communities, which applies to the study population, **n**_**0**_ is denotes the sample size in infinite communities (open communities), and **N** is denotes the size of the study population, where the researcher identified the study population from the official data issued by the department of student affairs at the secondary school, which is estimated from the reality of records of 3836 adolescent students.

The researcher used Smith's Equation to calculate a n_0_ as shown in Eq. (2) (Smith, 1983, p.90, p.90):

(2) $$n_{0}=\frac{Z^{2}\sigma^{2}}{e^{2}}$$

Where n_0_ is the sample size, z is the abscissa of the normal curve that cuts off an area α at the tails and the researcher determined it by 99% at the level of significance of 1%, which is estimated at ± 2.58., e is the desired level of precision (in the same unit of measure as the variance) which was determined by the researcher as only one degree, and σ is the variance of an attribute in the population.

By doing the calculations it was→: $n_{0}=\frac{{\left(2.58\right)}^{2}\times {\left(5,63\right)}^{2}\;=211}{{\left(1\right)}^{2}}$

The sample size in the study population can be calculated as follows: -

$$\begin{array}{l} n=\frac{n_{0}}{1+\frac{\left(n_{0}-1\right)}{\text{N}}}\\ \;=\frac{211}{1+\frac{\left(211-1\right)}{3836}}=200\;\text{adolescent}\;\text{students} \end{array}$$

**Figure 1:**
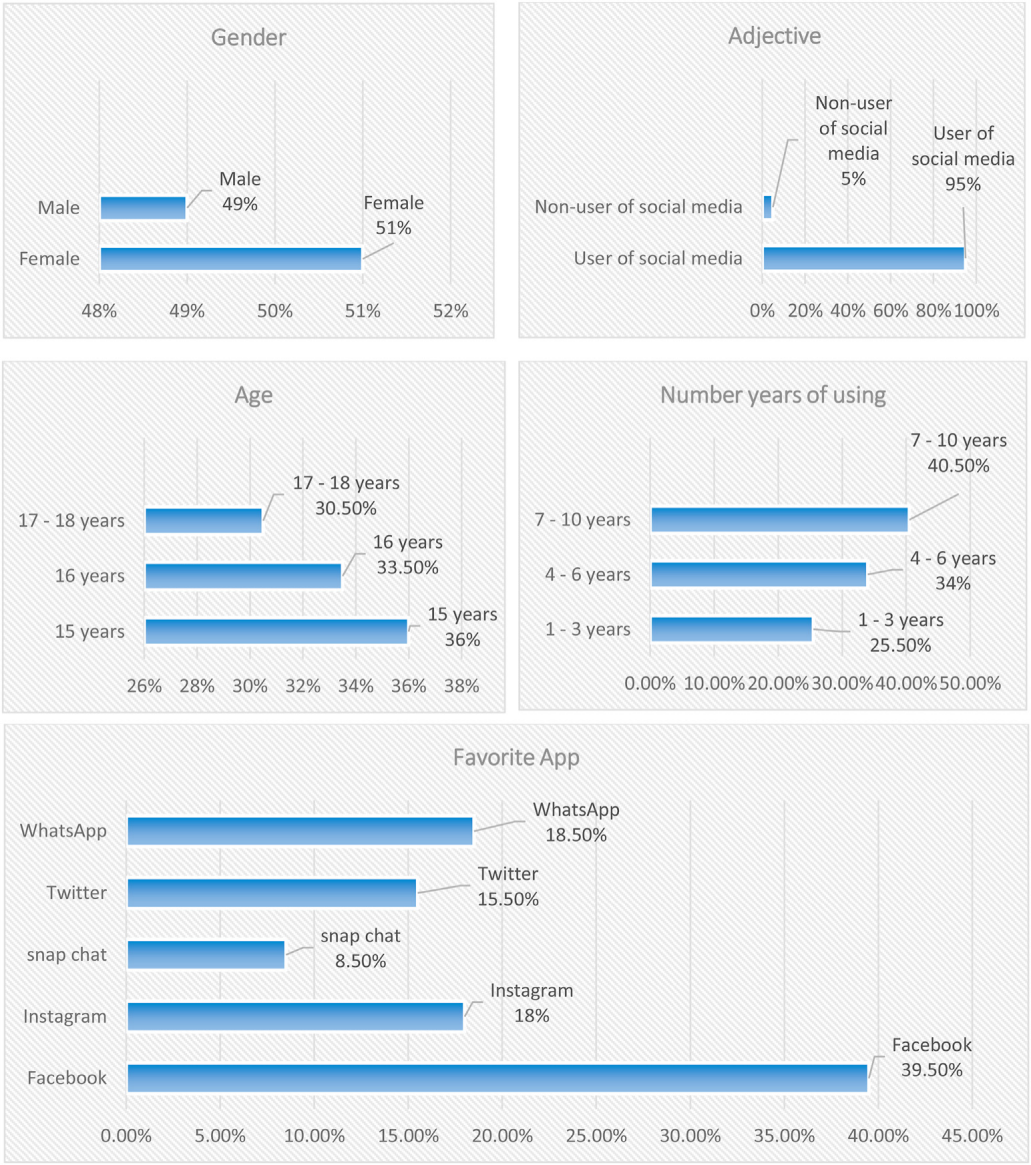
Demographic information on participants.

In the present research, the sample consisted of 200 adolescents (males 98 and females 102) form students the secondary stage. (see Table 1, Figure 1 shows the demographic information on participants).

**Table 1:** Demographic information on participants.

Variables	Statement	frequencies	percentage
Gender	Female	102	51%
	Male	98	49%
		**200**	**100%**
Adjective	User of social media	190	95%
	Non-user of social media	10	5%
	**Total**	**200**	**100%**
Age	15 years	72	36%
	16 years	67	33.5%
	17–18 years	61	30.5%
		**200**	**100%**
Number years of using	1–3 years	51	25.5%
	4–6 years	68	34%
	7–10 years	81	40.5%
	**Total**	**200**	**100%**
Favorite App	Facebook	79	39.5%
	Instagram	36	18%
	snap chat	17	8.5%
	Twitter	31	15.5%
	WhatsApp	37	18.5%
	**Total**	**200**	**100%**

In light of these results, it is clear that the percentage of females represents the highest rate at 51%, and followed Male at 49%. This converges with study (AL-Aag, 2013) which indicated that the proportion of males is less than or equal to females who use the internet is estimated at 50% (Any half of the total users). This may be justified because most of the girls in the Arab world after the end of their school day spend their spare time for long periods at home because they do not have the same space of freedom as the boys to spend fun time with their friends. This may be why girls are more attracted to using social media as an outlet to entertain themselves and socialize with many people online. The majority of adolescents are those in the age group of 15 years at 36%, followed by 16 years at 33.5%. It is clear that the majority of adolescents are users of social media on a large scale of 95%. This resulted from the ease of use and access of the internet, indoors, and outdoors through the various systems offered by telecommunications companies that are commensurate with the nature of the material possibilities of each individual. That corresponds with the study (Hinnawi, 2016), which confirmed the majority of students have at least one subscription on social media at the rate of 97%. besides, it is clear that the number years of using social media sites of adolescents is (7–10 years) by 40.5% of a user, followed by (4–6) by 34%. This is a significant indicator since these two stages represent the stage of early and middle childhood from the age of 8 years and above. Once the child reached adolescence, he was already addicted to the use of social media because he has spent most of his leisure time using it. In addition to, it turns out that the most popular social media sites frequented by adolescents are Facebook, at 39.5%, followed by WhatsApp, at 18.5%, followed by Instagram, at 18%. This is because these sites offer multiple features that increase the interaction between subscribers at no cost to the user, and it corresponds to the study (Kehinde and Adegbilero, 2016) the indicated that the adolescents are using Facebook in the rate of 93.48%, and also it corresponds to the study (Zawana, 2015) and study (Safar, 2017) which showed that a number of social media sites were the most used among the members They are in order: Facebook, WhatsApp, Twitter.

### 2.2 Ethical approval

This study was approved by the Scientific Research Ethics Committee of Ajman UAE. In addition to that, all participants provided informed consent before beginning the survey, besides, confirmation that the researcher complied with all relevant ethical regulations to maintain the confidentiality of the participants' information.

### 2.3 Study instrument

#### 2.3.1 Questionnaire

The researcher designed a new and innovative questionnaire that reflects the four main axes of James Marcia's theory of social identity (see 1.2. in 1. Introduction) in order to evaluate the impact of social media sites on the levels of social identity for adolescents. The questionnaire consists of 40 phrases, and the researcher managed the sincerity and reliability of the questionnaire as follows:

Validity of the questionnaire: The research tool was confirmed by the virtual validity method for the questionnaire by presenting it in its initial form with a list of study questions, to ten members of the teaching staff of universities, all of whom were doctorate holders in social work, sociology, psychology, and education. The content was adjusted according to their recommendations.

Reliability of the questionnaire: The researcher verified the reliability of the questionnaire by using the test-retest method. The questionnaire was applied to a small random sample consisting of 30 adolescents in secondary school, and fifteen days after the test was reapplied to the same sample of adolescents. After that, the Spearman correlation coefficient between the two applications was calculated, it is worth noting that the reliability coefficient was calculated according to Spearman's law of correlation coefficient as shown in Eq. (3): -

(3) $$r_{s}=1-\frac{6\left(\sum d^{2}\right)}{n\left(n^{2}-1\right)}$$

]In light of these results, the total reliability coefficient of 0.80 was considered appropriate for the purposes of this study as shown in Table 2, and the stability of the questionnaire is evident with a high confidence degree = 0.80 = 89%, It is a high coefficient, so the questionnaire has its validity, reliability and a high level of internal consistency.

**Table 2:** Shows the stability of the questionnaire and its variables.

Variables of the questionnaire	Rs
Level of "achievement" in the social identity of adolescents.	0.815
Level of "postponement" in the social identity of adolescents.	0.78
Level of "closure" in the social identity of adolescents.	0.775
Level of "dispersion" in the social identity of adolescents.	0.83
**TOTEL**	**0.80**

### 2.4 Data analysis measures

To find out the views of adolescents about the degree of the negative effects of the means of social communication on levels their social identity, a three-dimensional Likert scale is adopted as follows: agree (3), neutral (2), and disagree (1), as shown in Table 3 with the options used to evaluate counting periods.

**Table 3:** The evaluation of scale data based on the options of scale and score intervals.

Options	Scores	Score intervals
Agree	3	2.34–3.00
Neutral	2	1.67–2.33
Disagree	1	1.00–1.66

### 2.5 Methods of analyzing

The researcher used descriptive analysis to collect, analyze, and interpret the data for the study methodology, this is because this study falls under the descriptive research pattern aimed at describing and analyzing the variables of the study so that the researcher can obtain accurate data (Mohammed, 2012, p.48) and information depicting the reality of the situation (Mowaffaq, 2006, p55). Descriptive analysis is defined as a method of study and a systematic and objective way to explain and measure phenomena (Sandelowski, 1995, pp 372, 374). The description is then linked by comparison and interpretation to reach accurate results as to the nature of the dimensions of the social identity of adolescents and the extent of the negative effects of social media on them and determine the most popular social media used by adolescents, in addition to determining the differences between users and non-users of the areas of social identity associated with each of its four dimensions. This is in light of the monitoring, analysis, and interpretation of the data that was accessible from the study sample, extracting accurate conclusions and recommendations.

### 2.6 Analysis of statistics

The researcher used the Statistical Package for the Social Sciences (SPSS) analytical software for conducting the descriptive statistical analysis of data to analyze and interpret the data, in addition to some statistical coefficients were used to answer the study questions, which were (frequencies, percentage, arithmetic mean, and standard deviation) to characterize sample data. in addition to (T-test) and was used to study gender differences in social identity, as well as differences in the use of gender communication networks, as well as differences in the levels of social identity in terms of (achievement - postponement - closure - dispersion). and one-way ANOVA test to find out the significance of the differences between averages.

## 3 Results

### 3.1 Study findings related to RQ1

The question was: What are the negative effects of social media on the level of "achievement" in the social identity of adolescents from the perspective of social work?

**Figure 2:**
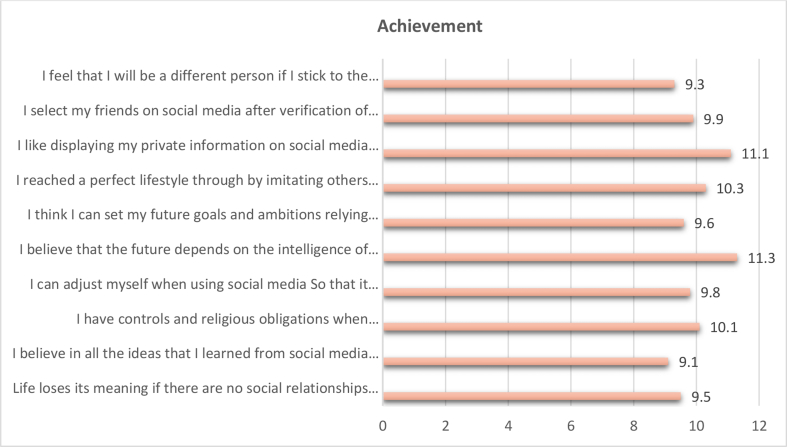
The negative effects of social media on the level of "achievement" in the social identity of adolescents.

The total weights, weighted relative weight, percentage of the negative effects of social media on the level of " achievement " in the social identity of adolescents were calculated by using the Questionnaire instrument and then arranging the negative effects of social media on the level of "achievement" according to their total weights, weighted relative weight, and percentage from High to low (see Table 4, Figure 2).

**Table 4:** The negative effects of social media on the level of "achievement" in the social identity of adolescents.

The negative effects of social media on the level of "Achievement"	Total weights	Weighted relative weight %	percentage %	Ranking
Life loses its meaning if there are no social relationships via social media more than real social relations in reality.	383	63.83	9.5	8
I believe in all the ideas that I learned from social media more than the ideas that I learned from my parents.	365	60.83	9.1	10
I have controls and religious obligations when communicating with the opposite sex via social media.	404	67.33	10.1	4
I can adjust myself when using social media So that it does not affect my natural roles in life to the fullest.	394	65.67	9.8	6
I believe that the future depends on the intelligence of the individual in how he uses the benefits of social media to delight himself more than serve society.	454	75.67	11.3	1
I think I can set my future goals and ambitions relying on the ideas transmitted by others through social media.	386	64.33	9.6	7
I reached a perfect lifestyle through by imitating others through social media.	415	69.17	10.3	3
I like displaying my private information on social media to all strangers.	446	74.33	11.1	2
I select my friends on social media after verification of their personalities and make sure that there are common aspects of thinking between us.	401	66.83	9.9	5
I feel that I will be a different person if I stick to the values transmitted to us contrary to our culture via social media without paying attention to the values and standards of our community.	372	62	9.3	9
**Total**	**4020**		**100%**	
**Weighted relative weight of the variable**	67%			
**Level of weight representation**	Middle

We note from the results that the negative effects of the social media on the level of "achievement” in the social identity of adolescents obtained a total weight of (4020), weighted relative weight of (67%). This indication is a medium, indicating that the level of impact is average. It is clear from the analysis that the phrase (I believe that the future depends on the intelligence of the individual in how he uses the benefits of social media to delight himself more than serve society) showed the highest percentage of all the negative effects of social media on the level of " achievement " in the social identity of adolescents, with total weights (454), Weighted relative weight (75.67), percentage of 11.3 % and Ranking (1).

### 3.2 Study findings related to RQ2

The question was: What are the negative effects of social media on the level of "postponement" in the social identity of adolescents from the perspective of social work?

**Figure 3:**
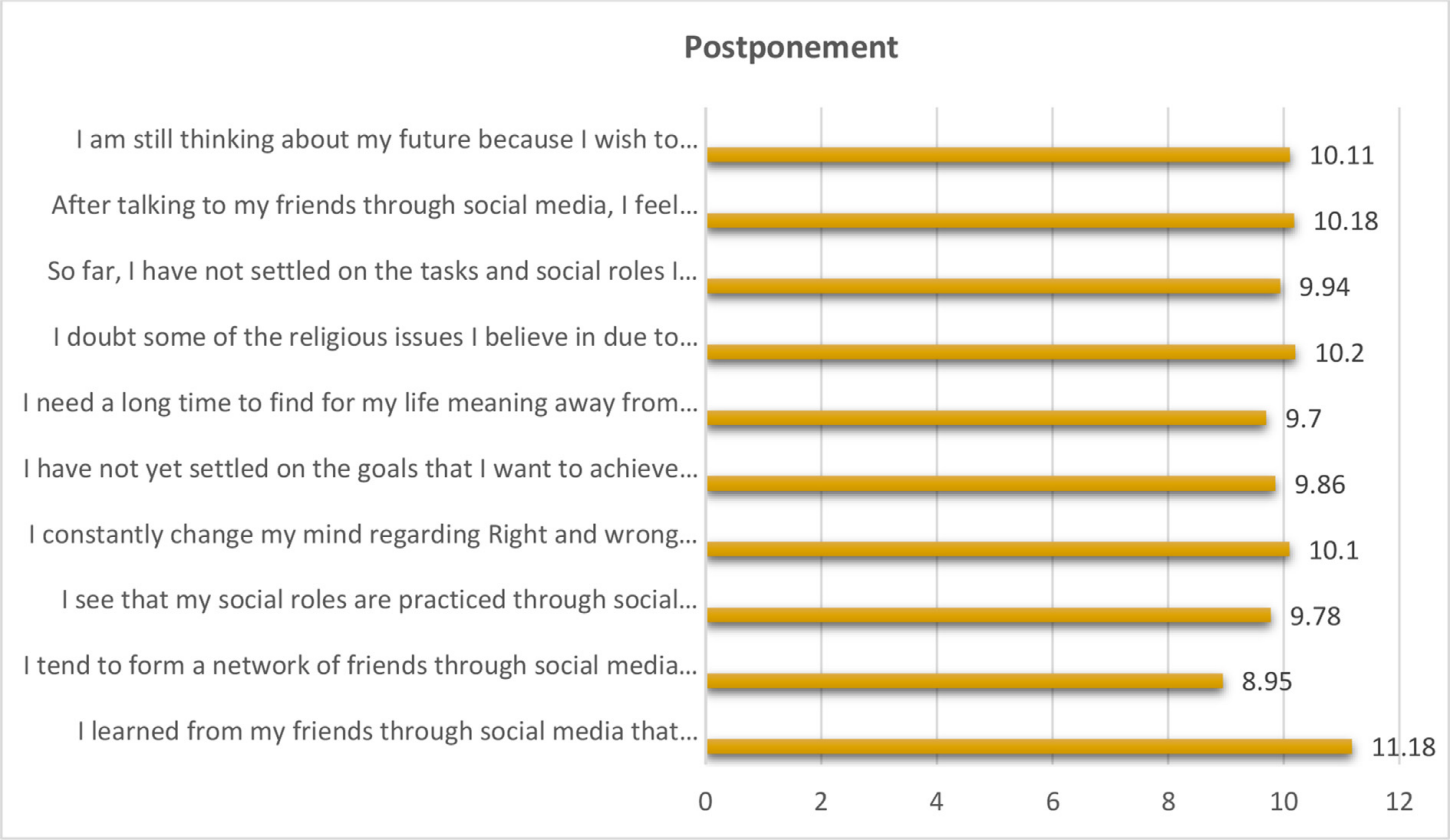
The negative effects of social media on the level of "postponement" in the social identity of adolescents.

The total weights, weighted relative weight, percentage of the negative effects of social media on the level of "postponement " in the social identity of adolescents were calculated by using the Questionnaire instrument and then arranging the negative effects of social media on the level of "postponement" according to their total weights, weighted relative weight, and percentage from High to low (see Table 5, Figure 3).

**Table 5:** The negative effects of social media on the level of "postponement" in the social identity of adolescents.

The negative effects of social media on the level of "Postponement"	Total weights	Weighted relative weight %	percentage %	Ranking
I learned from my friends through social media that achieving a self does not require logic in thinking and does not require speed in decision-making for any reason.	456	76	11.18	1
I tend to form a network of friends through social media because I don't know how to choose the best type of friends.	365	60.83	8.95	10
I see that my social roles are practiced through social media better even if some people criticize me for lack of interest or exaggerated delay.	399	66.5	9.78	8
I constantly change my mind regarding Right and wrong behaviors on social media because the times are changed.	410	68.33	10.1	5
I have not yet settled on the goals that I want to achieve in the future as a result of the continuous development in information technology on social media.	402	67	9.86	7
I need a long time to find for my life meaning away from social media.	396	66	9.7	9
I doubt some of the religious issues I believe in due to the different opinions of my friends on social media.	416	69.33	10.2	2
So far, I have not settled on the tasks and social roles I perform as a result of openness and modern methods on the social media.	405	67.5	9.94	6
After talking to my friends through social media, I feel confused about our social values and how useful it.	415	69.17	10.18	3
I am still thinking about my future because I wish to resemble the lifestyle of my friends on social media.	412	68.67	10.11	4
**Total**	**4076**		**100%**	
**Weighted relative weight of the variable**	67.93%			
**Level of weight representation**	Middle

We note from the results that the negative effects of the social media on the level of "postponement” in the social identity of adolescents obtained a total weight of (4076), weighted relative weight of (67.93%). This indication is a medium, indicating that the level of impact is average. It is clear from the analysis that the phrase (I learned from my friends through social media that achieving a self does not require logic in thinking and does not require speed in decision-making for any reason) showed the highest percentage of all the negative effects of social media on the level of " postponement " in the social identity of adolescents, with total weights (456), Weighted relative weight (76), percentage of 11.18 % and Ranking (1).

### 3.3 Study findings related to RQ3

The question was: What are the negative effects of social media on the level of "closure" in the social identity of adolescents from the perspective of social work?

**Figure 4:**
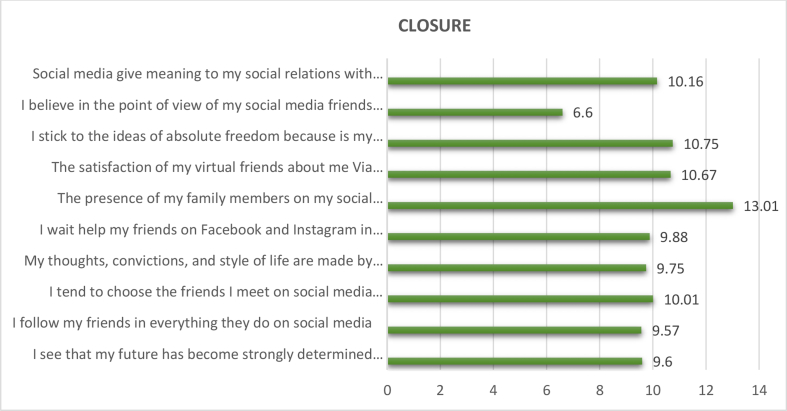
The negative effects of social media on the level of " closure " in the social identity of adolescents.

The total weights, weighted relative weight, percentage of the negative effects of social media on the level of " closure " in the social identity of adolescents were calculated by using the Questionnaire instrument and then arranging the negative effects of social media on the level of " closure " according to their total weights, weighted relative weight, and percentage from High to low (see Table 6, Figure 4).

**Table 6:** The negative effects of social media on the level of "closure" in the social identity of adolescents.

The negative effects of social media on the level of "Closure"	Total weights	Weighted relative weight %	percentage %	Ranking
I see that my future has become strongly determined by my relationships with friends in my virtual community Via social media.	374	62.33	9.6	8
I follow my friends in everything they do on social media.	373	62.17	9.57	9
I tend to choose the friends I meet on social media because I don't have to meet them face to face.	390	65	10.01	5
My thoughts, convictions, and style of life are made by the views of my friends on social media.	380	63.33	9.75	7
I wait help my friends on Facebook and Instagram in setting my goals in life.	385	64.17	9.88	6
The presence of my family members on my social media account is imposed on me, and I am inside me I don't agree on it.	507	84.5	13.01	1
The satisfaction of my virtual friends about me Via social media makes me feel comfortable.	416	69.33	10.67	3
I stick to the ideas of absolute freedom because is my way to get closer to others by raising the number of followers on social media.	419	69.83	10.75	2
I believe in the point of view of my social media friends regarding sexual intercourse.	257	42.83	6.6	10
Social media give meaning to my social relations with others through their acceptance of me and their interaction with me.	396	66	10.16	4
**Total**	**3897**		**100%**	
**Weighted relative weight of the variable**	64.95 %			
**Level of weight representation**	Middle

We note from the results that the negative effects of the social media on the level of "closure” in the social identity of adolescents obtained a total weight of (3897), weighted relative weight of (64.95 %). This indication is a medium, indicating that the level of impact is average. It is clear from the analysis that the phrase (The presence of my family members on my social media account is imposed on me, and I am inside me I don't agree on it) showed the highest percentage of all the negative effects of social media on the level of " closure " in the social identity of adolescents, with total weights (507), Weighted relative weight (84.5), percentage of 13.01% and Ranking (1).

### 3.4 Study findings related to RQ4

The question was: What are the negative effects of social media on the level of "dispersion" in the social identity of adolescents from the perspective of social work?

**Figure 5:**
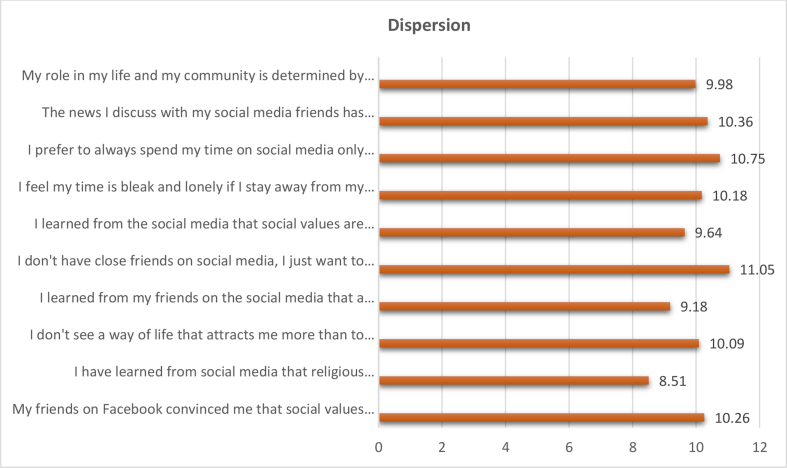
The negative effects of social media on the level of " dispersion " in the social identity of adolescents.

The total weights, weighted relative weight, percentage of the negative effects of social media on the level of " dispersion" in the social identity of adolescents were calculated by using the Questionnaire instrument and then arranging the negative effects of social media on the level of "dispersion" according to their total weights, weighted relative weight, and percentage from High to low (see Table 7, Figure 5).

**Table 7:** The negative effects of social media on the level of "dispersion" in the social identity of adolescents.

The negative effects of social media on the level of "Dispersion"	Total weights	Weighted relative weight %	percentage %	Ranking
My friends on Facebook convinced me that social values are a mask to reach personal interests.	417	69.5	10.26	4
I have learned from social media that religious commitment is not something important as long as I am comfortable with what I doing of action.	346	57.67	8.51	10
I don't see a way of life that attracts me more than to any other because of the events and problems that I read and discussed through social media.	410	68.33	10.09	6
I learned from my friends on the social media that a person lives his day and does not tire himself thinking about the future.	373	62.17	9.18	9
I don't have close friends on social media, I just want to be among the participants on the social media pages in order for me to feel the importance me of being in life.	449	74.83	11.05	1
I learned from the social media that social values are imposed on us and we are the only ones who carry them through life as a result of our society's culture.	392	65.33	9.64	8
I feel my time is bleak and lonely if I stay away from my virtual relationships with my friends on social media.	414	69	10.18	5
I prefer to always spend my time on social media only because I am not integrated into ideas and convictions with my family members.	437	72.83	10.75	2
The news I discuss with my social media friends has made me believe that life is difficult and tiring for many of us.	421	70.17	10.36	3
My role in my life and my community is determined by the experiences, including traumatic experiences, my friends bring to me via social media.	406	67.67	9.98	7
**Total**	**4065**		**100%**	
**Weighted relative weight of the variable**	67.75%			
**Level of weight representation**	Middle

We note from the results that the negative effects of the social media on the level of "dispersion” in the social identity of adolescents obtained a total weight of (4065), weighted relative weight of (67.75%). This indication is a medium, indicating that the level of impact is average. It is clear from the analysis that the phrase (I don't have close friends on social media, I just want to be among the participants on the social media pages in order for me to feel the importance me of being in life) showed the highest percentage of all the negative effects of social media on the level of " dispersion " in the social identity of adolescents, with total weights (449), Weighted relative weight (74.83), percentage of 11.05% and Ranking (1).

### 3.5 Study findings related to RQ5

The question was: Does the degree of awareness adolescents of the negative effects of social media on levels of their social identity vary according to gender, adjective, age, number years of using, and favorite app?

In order to answer the fifth research question of the study, the investigator measured the mean scores and standard deviations. In order to find out the importance of the variations between averages, the investigator then conducted an independent T-test and a one-way ANOVA test. In the following section, the findings are detailed.

#### 3.5.1 Gender

An independent sample test (T) was used by the researcher to determine the importance of the discrepancies between averages of adolescents' awareness of the negative impact of social media on their social identity levels. The results were measured by gender (see Table 8).

**Table 8:** Mean and SD by gender of the adolescent's responses.

Gender	N	Mean	Std. Deviation	T. Value	Sig. (tailed)	Sig. level
Female	102	2.98	0.809	-3.017	0.000∗	Significant
Male	98	3.87	0.645			

∗ Statistically significant at (α 0.05).

The results in Table 8 show that the computed value of (T) was (-3,017), which is greater than that of the table of (T). This implies that at the significance level of (0.000), which is less than the required statistical significance level (0.05), there are substantial differences between the mean value of male and female, where females are preferred over males.

#### 3.5.2 Adjective

An independent sample test (T) was used by the researcher to determine the importance of the discrepancies between averages of adolescents' awareness of the negative impact of social media on their social identity levels. The results were measured by adjective (see Table 9).

**Table 9:** Mean and SD by an adjective of the adolescent's responses.

Adjective	N	Mean	Std. Deviation	T. Value	Sig. (tailed)	Sig. level
User of networks	190	3.22	0.976	-4.019	0.000∗	Significant
Non-user of networks	10	5.01	0.412			

∗ Statistically significant at (α 0.05).

The results in Table 9 show that the computed value of (T) was (-4.019), which is greater than that of the table of (T). This implies that at the significance level of (0.000), which is less than the required statistical significance level (0.05), there are substantial differences between the mean value of Users of networks and Non-users of networks, where the User of networks are favored over the Non-user of networks.

#### 3.5.3 Age

Table 10. Shows the ANOVA one-way test results to evaluate the responses of the adolescent according to age.

**Table 10:** One-way ANOVA of the responses of adolescents by age.

		Sum of Squares	df	Mean Square	F	Sig. (tailed)	Sig. level
Age	Between Groups	4.000	3	1.900	2.721	0.178∗	Not Significant
	Within Groups	107.192	129	.652			
	Total	111.192	132				

∗Statistically significant at (α 0.05).

In Table 10. There are no statistically significant variations in the viewpoints of teenagers according to the age variable at 0.178, which is greater than the required statistical significance level of 0.05.

#### 3.5.4 Number years use of social media

Table 11. Shows the ANOVA one-way test results to evaluate the responses of the adolescents according to number years use of social media.

**Table 11:** One-way ANOVA of the responses of adolescents by number years use of social media.

		Sum of Squares	df	Mean Square	F	Sig. (tailed)	Sig. level
Number years use of social media	Between Groups	4.000	3	1.430	1.503	0.142∗	Not Significant
	Within Groups	107.192	129	.564			
	Total	111.192	132				

∗Statistically significant at (α 0.05).

In Table 11. There are no statistically significant variations in the viewpoints of teenagers according to the number years use of social media variable at 0.142, which is greater than the required statistical significance level of 0.05.

#### 3.5.5 Favorite app

Table 12. Shows the ANOVA one-way test results to evaluate the responses of the adolescents according to favorite app.

**Table 12:** One-way ANOVA of the responses of adolescents by favorite app.

		Sum of Squares	df	Mean Square	F	Sig. (tailed)	Sig. level
Favorite App	Between Groups	4.000	3	1.099	1.103	0.123∗	Not Significant
	Within Groups	107.192	129	.477			
	Total	111.192	132				

∗Statistically significant at (α 0.05).

In Table 12. There are no statistically significant variations in the viewpoints of teenagers according to the favorite app variable at 0.123, which is greater than the required statistical significance level of 0.05.

## 4 Discussion

This study aimed to identify the negative effects of social media on "levels" the social identity of adolescents in the secondary stage from the perspective of social work, the results showed that the negative effects of the social media on the level of "achievement” in the social identity of adolescents obtained a total weight of (4020), weighted relative weight of (67%). This indication is a medium, indicating that the level of impact is average. The obtained results, are shown in Table 4, and concern the extent negative effects of social media on the level of "achievement" of the social identity of adolescents. We have noticed that most adolescent responses are indicating a lack of interest in the effective role in normal life, and rather transforming themselves into being united with an electronic world where the quality of values and principles are different from that of previous generations. Here we see that some teenagers are trying to have an entity and a role but many of them cannot because of the dangerous and influential role of the internet which depends on dazzling and attracting the longest number of hours in front of social media. Although, James Marcia believes this level is the most mature level of identity because it integrates and develops the growth of the personality of the teenager through the development and identification of tasks and pledges clearly and specifically. However, the adolescent cannot reach a strong level due to the fact that many of them are driven towards the negative impact of these sites. This corresponds with a study (Bu-Abdullah, 2016) which indicated to emphasized that the use of the Internet leads to a lack of interest in the effective role in their life and Delayed level of academic achievement in adolescent pupils as well, the obtained results, are shown in Table 5, and concern the negative effects of the social media on the level of "postponement” in the social identity of adolescents obtained a total weight of (4076), weighted relative weight of (67.93%). This indication is a medium, indicating that the level of impact is average. We note from the results and It is clear from the analysis that the negative effects of social media on the level of "Postponement" of the social identity of adolescents indicating the inability of an adolescent to come up with a clear idea of the things he wants. Besides, his goals in life are almost clear but he cannot make decisions about them, he is a person whose character is fluctuating and contradictory for fear of taking responsibility or committing to specific promises to himself or the community around him. This agrees with James Marcia's opinion that the teenager in this rank is in a period of exploration and has unclear and vague commitments. and has not set his position on many of his life issues This corresponds with a study (Bu-Abdullah, 2016) which indicated to emphasized, the availability of the Internet inside the home increases the duration of use of the adolescent to a social network, and this too leads postponing adolescents for many of the goals in addition to the Fluctuation in opinion and inability to make clear decisions.

Results also showed in Table 6, that the negative effects of the social media on the level of "closure” in the social identity of adolescents obtained a total weight of (3897), weighted relative weight of (64.95 %). This indication is a medium, indicating that the level of impact is average. Moreover, the obtained results, as shown in Table 5, and concern the extent the negative effects of social media on the level of "closure" of the social identity of adolescents indicating that the adolescent is ineffective and awaits solutions and results from others, whether power, friends or society. This result reflects the extent of the turbulence experienced by adolescents with their lack of self-confidence due to lack of ability to choose and lack of self-confidence, whether at the future social level or at the religious level and that he cannot make a decision or take responsibility. James Marcia expresses this rank that the teenager does not have clear and specific commitments, but takes them ready from his parents or those around him. This contradicts the study (Hattat, 2014) which indicated that social networks reduce the degree of social isolation, but, the exact scores and percentages reached by the current study prove that social networking sites have a significant negative impact that leads to more of closure with their lack of self-confidence due to lack of ability to choose and lack of self-confidence as well, the obtained results, are shown in Table 7, and concern the negative effects of the social media on the level of "dispersion” in the social identity of adolescents obtained a total weight of (4065), weighted relative weight of (67.75%). This indication is a medium, indicating that the level of impact is average. We note from the results and It is clear from the analysis that the negative effects of social media on the level of "dispersion" of the social identity of adolescents, indicating the adolescent is less accepting of himself and his community. He sees himself as "inferior", making his thoughts and behaviors immature. In addition, he is also closer to the character of aggressiveness and may evolve to be psychopathic. This is consistent with James Marcia's assertion in his theory that the rank of "dispersion" of the lowest ranks of identity and it characterized that teenager does not have clear commitments and does not try to discover other options or alternatives and fails to adhere to a fixed ideology. This contradicts the study (Hattat, 2014) which indicated that social networks reduce lack of concentration but the accurate scores and percentages reached by the present study prove that social media have a significant negative impact that leads to more distractions, anxiety, lack of concentration and dispersion.

On the other hand, the obtained results, as shown in Tables 8 and 9, 10, 11, and 12 pertained to whether the degree of awareness adolescents of the negative effects of social media on levels of their social identity vary according to gender, adjective, age, number years of using, and favorite app. The results indicated that the degree of adolescent's awareness varies according to gender and adjective, with females being more aware of the negative effects of social media than males. maybe due to the fact that females' adolescents are more fearful and cautious about themselves as a result of socialization since childhood started and keener because the amount of accountability of parents to their daughter in the Arab world for the mistakes she makes is more severe and violent than the boy, which is why the girl is more cautious in her relationships with others through social media. It is also worth noting no statistically significant differences in adolescent's awareness were found based on the variables of age, number years of using, and favorite app.

## 5 Conclusions

**Figure 6:**
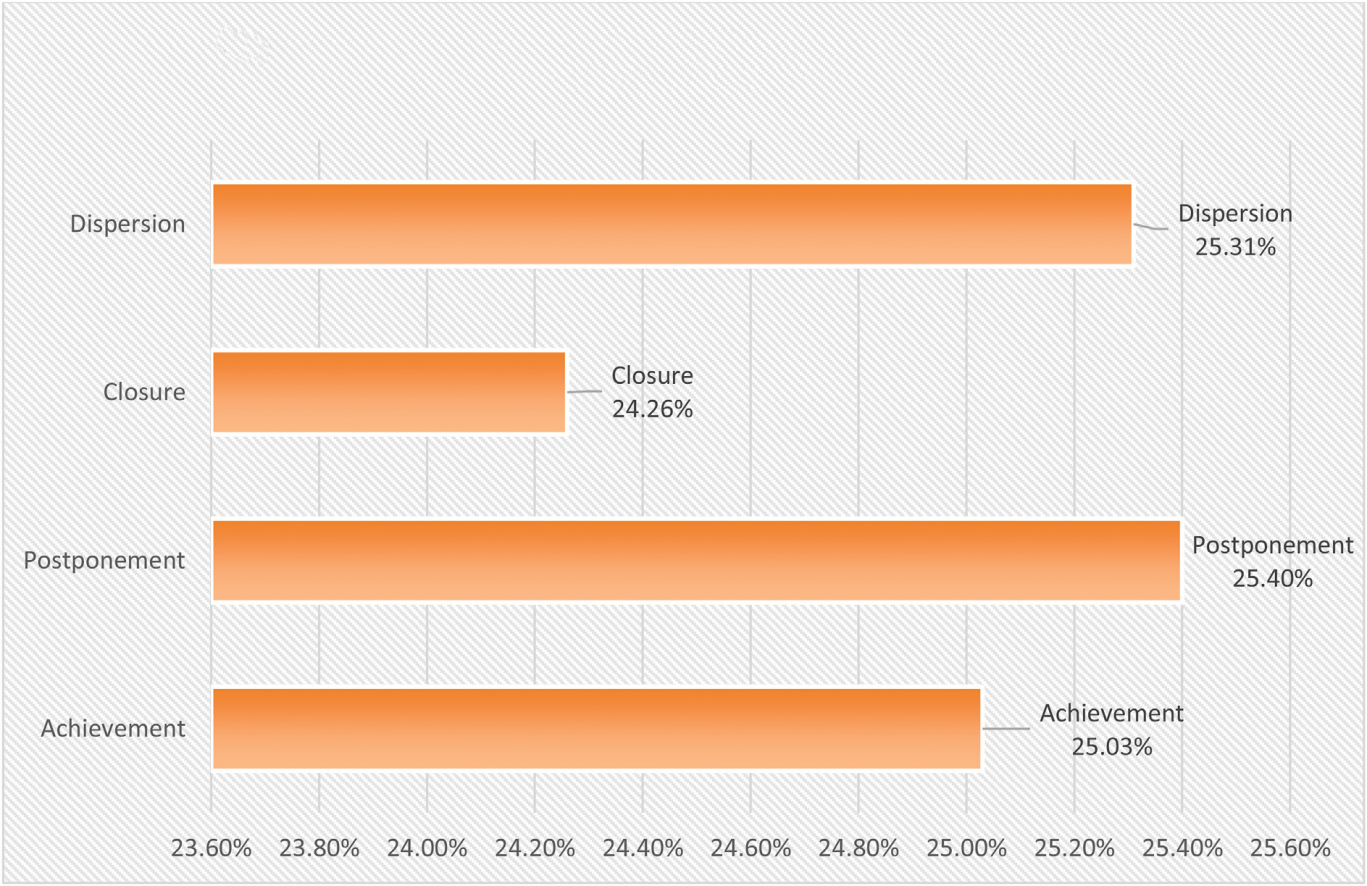
Ranking levels of the social identity of adolescents after all negative effects of social media on it.

From the results above, we can conclude that the value of all negative effects of social media on "levels" the social identity of adolescents from the perspective of social work came to a total weight of (16058), weighted relative weight of (66.9%). This indication is a medium, indicating that the level of impact is average for all negative effects of social media on the levels of social identity in adolescents. It ranked first "Postponement level" at 25.4%, It is followed by the ranked second “Dispersion level" at 25.31%, Then came third place "Achievement level" at 25.03%, Finally in fourth place "Closure level" at 24.26% (see Table 13, Figure 6).

**Table 13:** Ranking levels of the social identity of adolescents after all negative effects of social media on it.

Levels of social identity of adolescents	Total weights	Percentage %	Ranking
Achievement	4020	25.03%	**3**
Postponement	4076	25.4%	**1**
Closure	3897	24.26%	**4**
Dispersion	4065	25.31%	**2**
**Total**	**16058**	**100%**	
**Weighted relative weight of the variable**	66.9%		
**Level of weight representation**	Middle

### 5.1 In light of this, we reach an important conclusion, which is that

It is necessary taking serious measures from the family, school, and institutions that care for the family and children to pay attention to how to face negative effects of social media on social identity to children and adolescents. Besides working to encourage children and adolescents do not get lost their time and take the largest part of their free time in practicing sports and cultural activities. That corresponds with the study (Hamdi, 2018) which was emphasized in it that the most important motives for the use of social networking sites are entertainment for loss of time.

In addition to another important conclusion is train parents to help their children to make the best use of these sites so as not to be exposed to problems resulting from open communication without restrictions. and this corresponds to a study (Bu-Abdullah, 2016) which was emphasized that parents should know the programs which watching it their teenager child is on the Internet to guide them to useful things on social networks. Hence, the researcher presents her following recommendations.

## 6 Recommendations

Urge parents to follow their children continuously and guide them in the use of social networking sites.

Educating children about the need to observe the privacy of their information and data, so that it is not accessible to everyone, including strangers.

Educating the awareness of children not to accept video conversations or written conversations or requests for friendship from strangers.

Educating children's awareness of the need to not display their own pictures in public so as not to be copied by strangers and exploited inappropriately.

Urge children to inform their parents of any threat or blackmail they may face from anyone on the internet.

Urge parents to fill the leisure time of their children by encouraging them to practice a hobby or sport they love.

Urge parents not to excessively pamper their children or give them extra money so as not to spoil them.

Increase educational institution awareness seminars for students, giving information on the pros and cons of social networking sites.

Urge parents to establish a bridge of communication between them and their children and follow the method of persuasion, and not intimidation, when adapting their child's behavior on the internet.

## Declarations

### Author contribution statement

W. Elsayed: Conceived and designed the experiments; Performed the experiments; Analyzed and interpreted the data; Contributed reagents, materials, analysis tools or data; Wrote the paper.

### Funding statement

This research did not receive any specific grant from funding agencies in the public, commercial, or not-for-profit sectors.

### Data availability statement

Data will be made available on request.

### Declaration of interests statement

The authors declare no conflict of interest.

### Additional information

No additional information is available for this paper.

## References

[bib1] Abdul Jalil M.A. (2011). How communication contributed to weakening Social customs and traditions and reduced Social relations. Ninth Doha Conference for Interfaith Dialogue, 26 October.

[bib2] Abu Arad S.,B.,A. (2008). Leisure and Recreation in the Life of Muslim Youth (Concept and Practice), Saudi Arabia.

[bib3] AL-Aag H.N. (2013). The Use of the Internet in the Study and its Relationship to Motivation for Learning in Adolescents (12-14 years).

[bib4] Al-Ghamdi H.A.F. (2001). A relationship that constitutes the identity of Alana with moral thinking in a sample of males in adolescence and youth, Egypt. Egypt. J. Psychol. Stud..

[bib5] Al-Hafian F. (2004). Language and identity: problems of concepts and controversy of relationships. Al-Tasamoh J. Amman.

[bib6] Al-Jazi H. (2018). What Is Twitter and How to Use it, Egypt.

[bib7] Al-Mu'ti H. (2016).

[bib8] Al-Murshidi E.H.A. (2007). The Development of Understanding the Identity of Adolescents and its Relationship with Social Interaction.

[bib9] Al-Obaidi I. (2019). Social media and its Impact on Society, Egypt.

[bib10] AL-Oubli T.N.M. (2011). Psychometric Characteristics of the Scale of Identity and Crisis Crises for Adolescents in Housing Institutions, Egypt.

[bib11] Al-Shammasi E.M.M. (2006). General Education and Philosophy of Education.

[bib12] Al-Shareef H. (2014). A Collaborative Access Control Model for Shared Items in Online Social Networks.

[bib13] Al-Zu'bi A.M. (2001). Psychology of Childhood and Adolescence.

[bib14] Ali L.I. (2007). Ranks of Social and Ideological Identity and Their Relationship Psychological Alienation ", Unpublished Master Thesis, Supervised.

[bib15] Ali A. (2019).

[bib16] Asiri A. (2004). A Relationship that Constitutes the Identity of the Ego with Both the Concept of Self and harmony Psychosocial and General, Unpublished Master Thesis, Supervision.

[bib17] Asur S., Huberman B. (2010). Predicting the future with social media. Paper Presented at the Web Intelligence and Intelligent Agent Technology (WI-IAT), 2010.

[bib18] Awad R.A. (2013). The Effects of Using Social Media Sites on the Educational Achievement of Children in Tulkarm Governorate from the Perspective of Housewives.

[bib19] Bailey O.G., Camerez B., Cairo O.A. (2009). Understanding alternative media. Translation: Islah.

[bib20] Bakkar A. (2010). The Teenager How to Understand it and How to Direct it.

[bib21] Bouchey H.A., Furman W., Adams G., Berzonsky M.D. (2013). Dating and romantic experiences in adolescence. Blackwell Handbook of Adolescence.

[bib22] Bu-Abdullah S. (2016). Internet Uses and Their Impact on Students Adolescents “Secondary Field Study of Khadr Ramadan Omash-Biskra”, Algeria, Master Thesis.

[bib23] Cillessen A.H.N., Rose A.J. (2008). Understanding Popularity in the Peer System, Current Directions in Developmental Psychology.

[bib24] Cochran W.G. (1963). Sampling Techniques.

[bib25] Dawaq H. (2016). Religion and Identity between the Narrow Belonging and the Capacity of Creativity, Algeria.

[bib26] Erikson Erik (1994). Identity and the Life Cycle Paperback - April 17, New York - London.

[bib27] Hamdi M.A. (2018). University Youth Dependence on Social Media for Access to Information “A Survey Study at the University of Tabuk, Saudi Arabia”, Amman, Jordan, Master Thesis.

[bib28] Hamed D. (2018). WhatsApp, Egypt.

[bib29] Hattat M. (2014). Psychosocial Problems of School-Age Adolescents Internet Users, Algeria, Master Thesis in Mental Health and School Adaptation.

[bib30] Hayaty S. (2018). When Facebook Was Founded, Cairo.

[bib31] Hinnawi M.R. (2016). Uses of Middle Teen Students for Networks Social Networking in Nablus City Schools in Palestine, Alam Journal.

[bib32] Hosni A. (2011). The Impact of Social Networking Sites in the Development Youth Social Responsibility.

[bib33] Ito M., Horst H., Bittani M., boyed d (2008). Living and Learning with New Media: Summary of Findings from the Digital Youth Project.

[bib34] Kehinde F., Adegbilero L. (2016).

[bib35] Khader M. (2018).

[bib36] Laith W. (2011). The Impact of Facebook on Self-Esteem Among Youth in Tulkarm Governorate.

[bib37] Levesque Roger J.R. (2011). Encyclopedia of Adolescence.

[bib38] Mansour (2014). The role of social media in meeting the needs of Jordanian university youth, Jordan, Jordan. J. Soc. Sci..

[bib39] Marcia J.E. (1966). Development and validation of ego-identity status. J. Pers. Soc. Psychol..

[bib40] Marcia J.E. (1980). Identity in adolescence. Handb. Adole. Psychol..

[bib41] Marcia James E. (2011). Ego Identity: A Handbook for Psychosocial Research Softcover Reprint of the Original.

[bib42] Mashaal T. (2018). What Is Instagram.

[bib43] Mitchell Alex J., Hussain Shahana, Leaver James, Rajan Chandhini, Jones Andrew, Malcolm Natasha, Tim Coats (2016). Is there a difference between hospital-verified and self-reported self-harm? Implications for repetition. Gen. Hosp. Psychiatr..

[bib44] Mitchell Alex J., Yadegarfar Motahare, Gill John, Stubbs Brendon (2016). Case finding and screening clinical utility of the Patient Health Questionnaire (PHQ-9 and PHQ-2) for depression in primary care: a diagnostic meta-analysis of 40 studies. BJPsych open.

[bib45] Mohamed M. (2019). Definition of Facebook, Egypt.

[bib46] Mohammed M.M.S. (2012). Educational Measurement and Evaluation.

[bib47] Mohsen H.H. (2018). The Nature of Social Identity in the Light of Sociological Theories. https://annabaa.org/arabic/authorsarticles/16219.

[bib48] Morelli A.O., Zupanick C.E. (2020). CHILD DEVELOPMENT THEORY : ADOLESCENCE.

[bib49] Mowaffaq A. (2006). Fundamentals of Scientific Research, Jordan, Amman.

[bib50] Muzayd B.M. (2012). Virtual Communities as an Alternative to Real Societies/Faces Faces as a Model.

[bib51] Nomar M. (2012). Use and Impact of Social Networking Sites in Social Relations with the App a Sample of Users Facebook in Algeria.

[bib52] O'Keeffe G.S., Clarke-Pearson K. (2011). The impact of social media on children, adolescents, and families. Pediatrics.

[bib53] Rajah I. (2019).

[bib54] Safar A.M. (2017). The Role of Social Networks in the Consolidation of the Values of Citizenship from the Perspective of the Omani Youth, Amman, Jordan, Master Thesis.

[bib55] Salim K. (2008). Culture of Social Media and Local Communities.

[bib56] Salima H., Fayza H. (2014). The impact of using social media on the psychological identity of secondary school students.

[bib57] Sandelowski M. (1995). Qualitative analysis: what it is and how to begin. Res. Nurs. Health.

[bib58] Smith M.F. (1983). Sampling Considerations in Evaluating Cooperative Extension Programs. Florida Cooperative Extension Service Bulletin PE-1.

[bib59] Steinberg L. (2002).

[bib60] Zain Al Abdeen F. (2013). The Impact of Social Media on Children, Adolescents and Families, Laws of O'Keefft Schragen and Kathleen Clark-Pearson.

[bib61] Zawana A.I. (2015). The Degree of Using Social Networks as a Tool for Learning and Learning Among Jordanian university Students and the Achieved Satisfaction, Amman, Jordan, Master Thesis.

